# A Twisted Presentation: Adult Intestinal Malrotation Complicated by Caecal Volvulus and Perforation

**DOI:** 10.7759/cureus.115433

**Published:** 2026-08-30

**Authors:** Ekansh Hemnat Singh, Mohammad Saquib Ali, Abhinav Sengar

**Affiliations:** 1 General Surgery, Ganesh Shankar Vidyarthi Memorial Medical College, Kanpur, IND; 2 Gastrosrugery, Ganesh Shankar Vidyarthi Memorial Medical College, Kanpur, IND

**Keywords:** bowel perforation, caecal volvulus, emergency laparotomy, intestinal malrotation, ladd’s bands

## Abstract

Intestinal malrotation is a congenital anomaly caused by incomplete rotation and fixation of the midgut during embryological development. Although it is usually diagnosed in infancy, rare adult cases may present with acute intestinal obstruction or perforation. Cecal volvulus is an uncommon but potentially life-threatening complication of malrotation. We report a 35-year-old woman who presented with severe abdominal pain, progressive distension, and absolute constipation for two days. Clinical examination revealed generalized peritonitis with absent bowel sounds, and an erect radiograph demonstrated free gas beneath both hemidiaphragms, indicating hollow-viscus perforation. Because of generalised peritonitis mandating urgent operation, she proceeded directly to emergency laparotomy without cross-sectional imaging. Intraoperatively, a cecal volvulus was identified in association with intestinal malrotation and Ladd’s bands, together with a single perforation on the anterior wall of the ascending colon. The involved cecum and ascending colon were markedly distended and venously congested from the closed-loop obstruction but showed no frank ischemia or gangrene, and bowel color improved after detorsion. Ladd’s bands were divided, the volvulus detorsed, the perforation repaired primarily in two layers, and a defunctioning loop ileostomy fashioned. The patient had an otherwise uncomplicated postoperative recovery and was discharged on postoperative day seven. The loop ileostomy was reversed at three months, and at six months the patient was well, asymptomatic, and passing normal stool without evidence of recurrence. This report highlights the importance of considering congenital malrotation in adults presenting with an acute abdomen and discusses the rationale for bowel-preserving surgery when viability is retained.

## Introduction

Intestinal malrotation results from incomplete rotation and fixation of the midgut during fetal development. Although it characteristically presents in infancy, adult presentations are rare. Symptomatic malrotation in adults is uncommon and accounts for only a small proportion of all cases, and its nonspecific presentation frequently leads to diagnostic delay [1]. Cecal volvulus - a torsion of the cecum, ascending colon, and terminal ileum - is an uncommon cause of large-bowel obstruction and represents roughly one-third of colonic volvulus cases in reported Western series, with a relatively greater share in younger patients [2].

The coexistence of intestinal malrotation and cecal volvulus in an adult is extremely rare and poses significant diagnostic and therapeutic challenges. When it occurs, it may culminate in bowel ischemia, necrosis, and perforation, requiring urgent surgical intervention [3]. During normal embryonic development, the midgut undergoes a 270-degree counterclockwise rotation around the superior mesenteric artery. Failure of this process leads to abnormal bowel position and a narrow mesenteric pedicle, predisposing to volvulus and obstruction. Ladd’s bands - aberrant fibrous peritoneal folds that typically extend from the caecum and right colon across the duodenum to the lateral abdominal wall and retroperitoneum - may additionally cause duodenal or colonic compression [4].

In adults, symptoms may be intermittent or vague, including chronic abdominal pain, nausea, and bloating. In the acute setting, patients may present with features of intestinal obstruction, such as abdominal pain, distension, vomiting, and non-passage of stool and flatus [5]. Because of this variability, the diagnosis is frequently delayed unless a high index of suspicion is maintained [6].

Computed tomography (CT) of the abdomen is the preferred cross-sectional imaging modality for evaluating suspected intestinal malrotation in adults. It can demonstrate an abnormally positioned duodenojejunal junction, small bowel on the right and colon on the left, inversion of the superior mesenteric artery-vein relationship, and, in volvulus, the classic mesenteric “whirl sign” [7]. However, in patients with generalized peritonitis and radiographic evidence of pneumoperitoneum who require immediate operative intervention, CT may reasonably be omitted when obtaining cross-sectional imaging would delay definitive surgical management. In such circumstances, the underlying anatomical abnormality may be diagnosed intraoperatively, as in the present case [6].

Management requires prompt surgical intervention, and Ladd’s procedure remains the cornerstone: counterclockwise detorsion of the volvulus, division of Ladd’s bands, broadening of the mesenteric base and repositioning of the bowel to reduce future risk. Where ischemia or perforation is present, resection with or without a temporary stoma may be required; where the bowel remains viable, a bowel-preserving repair with proximal diversion is an accepted alternative [8]. Early recognition and surgical management are essential to prevent fatal outcomes [9,10].

The aim of this report is to describe the rare coexistence of adult intestinal malrotation and caecal volvulus complicated by colonic perforation, to discuss the mechanism and site of perforation, and to outline the rationale for bowel-preserving surgery.

## Case presentation

A 35-year-old woman presented to the emergency department with severe abdominal pain, progressive distension, and absolute constipation of two days’ duration. There was no history of vomiting, previous abdominal surgery, or chronic illness. She was gravida 2, para 2, with a body mass index of 24.5 kg/m², and was classified as ASA physical status III E. On examination, she was mildly dehydrated but hemodynamically stable. The abdomen was markedly distended, diffusely tender with guarding, and bowel sounds were absent, consistent with generalised peritonitis.

Routine laboratory investigations (Table 1) showed leukocytosis (16.5 × 10⁹/L) with elevated inflammatory markers - C-reactive protein (CRP) 145 mg/L and serum lactate 2.8 mmol/L - together with mild elevation of serum creatinine (1.4 mg/dL), aspartate aminotransferase/serum glutamic-oxaloacetic transaminase (AST/SGOT) (43 IU/L), and alanine aminotransferase/serum glutamic-pyruvic transaminase (ALT/SGPT) (41 IU/L). These findings were consistent with sepsis and bowel compromise, with pre-renal azotaemia and mild transaminitis; hemoglobin and serum electrolytes were within normal limits.

**Table 1:** Laboratory investigations at admission Values outside the reference range are indicated in bold and with an upward arrow (↑). AST, aspartate aminotransferase; SGOT, serum glutamic-oxaloacetic transaminase; ALT, alanine aminotransferase; SGPT, serum glutamic-pyruvic transaminase; CRP, C-reactive protein

Investigation	Value at admission	Reference range
Haemoglobin	13.2 g/dL	12.0–15.0
Total leucocyte count	16.5 ×10⁹/L (↑)	4.0–11.0
Serum creatinine	1.4 mg/dL (↑)	0.6–1.2
AST (SGOT)	43 IU/L (↑)	< 40
ALT (SGPT)	41 IU/L (↑)	< 34
Serum sodium / potassium	136 / 3.8 mmol/L	135–145 / 3.5–5.0
CRP	145 mg/L (↑)	< 5 mg/L
Serum lactate	2.8 mmol/L (↑)	0.5–2.2 mmol/L

An erect chest and abdominal radiograph demonstrated free gas beneath both hemidiaphragms, indicating hollow-viscus perforation, together with grossly dilated, gas-filled bowel loops. Given the generalized peritonitis and unequivocal pneumoperitoneum, emergency laparotomy was indicated, and CT was not performed because obtaining cross-sectional imaging would have delayed definitive operative management. The underlying intestinal malrotation was therefore diagnosed intraoperatively. The patient was resuscitated and received intravenous ceftriaxone (2 g) and metronidazole (500 mg); the interval from emergency-department presentation to surgical incision was 2.5 hours.

After resuscitation with intravenous fluids, nasogastric decompression and broad-spectrum antibiotics, the patient underwent emergency exploratory laparotomy. Intraoperatively, a cecal volvulus was identified with a single perforation on the anterior wall of the ascending colon. The small-bowel loops lay to the right and the colon to the left, with Ladd’s bands extending from the cecum and right colon across to the retroperitoneum, findings consistent with intestinal malrotation. The intraoperative anatomical configuration, including the distended and twisted cecum, malrotated bowel loops, and Ladd’s bands, is shown in Figure 1.

**Figure 1:**
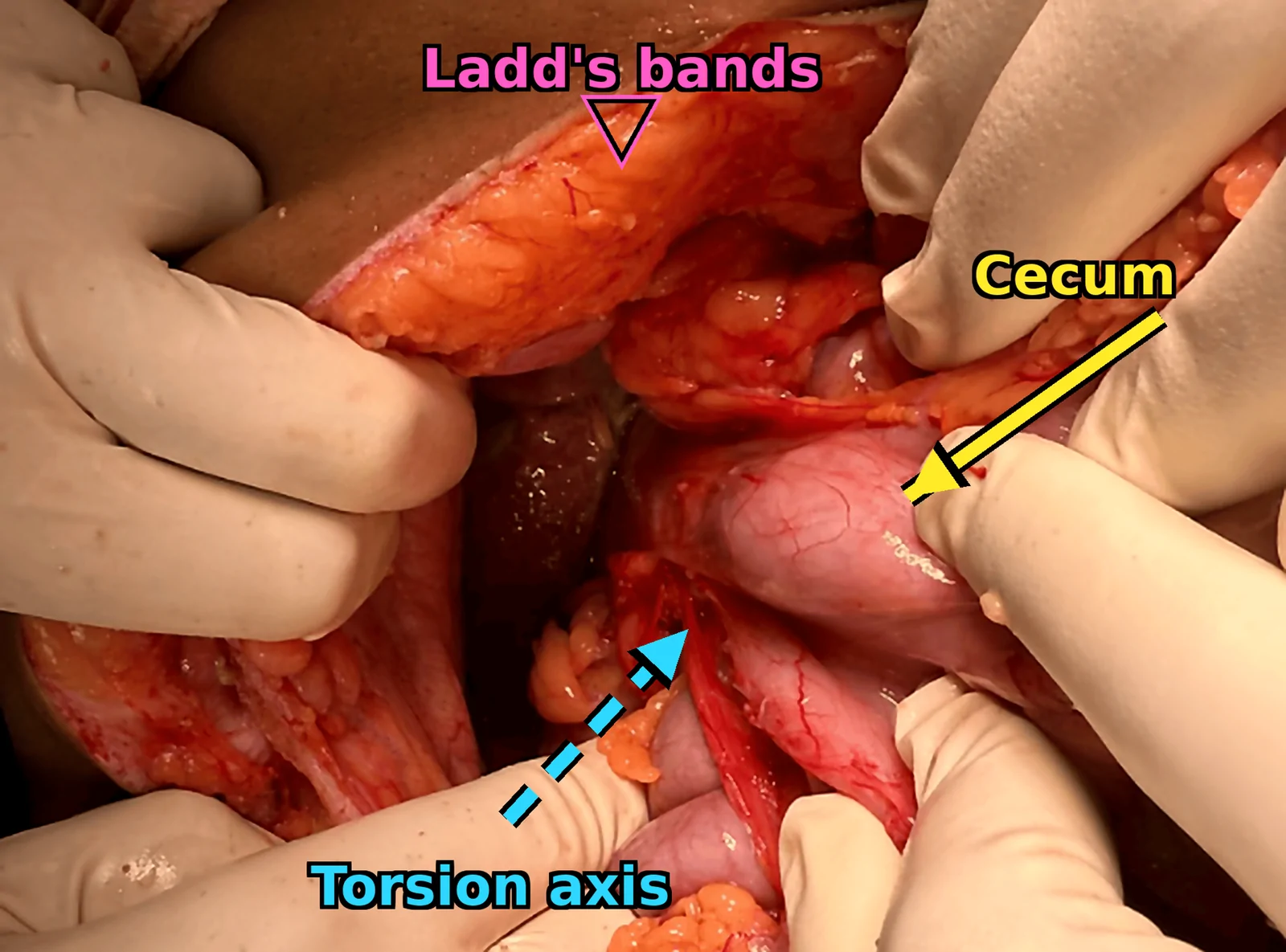
Intraoperative view showing the caecum, Ladd’s bands, and axis of torsion Intraoperative photograph showing a grossly distended and twisted caecum with malrotated bowel loops and fibrous Ladd’s bands. Solid arrow: distended cecum; dashed arrow: axis of torsion; arrowhead: Ladd’s band

The involved cecum and ascending colon were markedly distended and venously congested owing to the closed-loop obstruction, but there was no frank ischemia, gangrene, or established necrosis. The marked distension of the cecum and ascending colon is shown in Figure 2. The twisted mesenteric pedicle at the root of the volvulus is shown in Figure 3.

**Figure 2:**
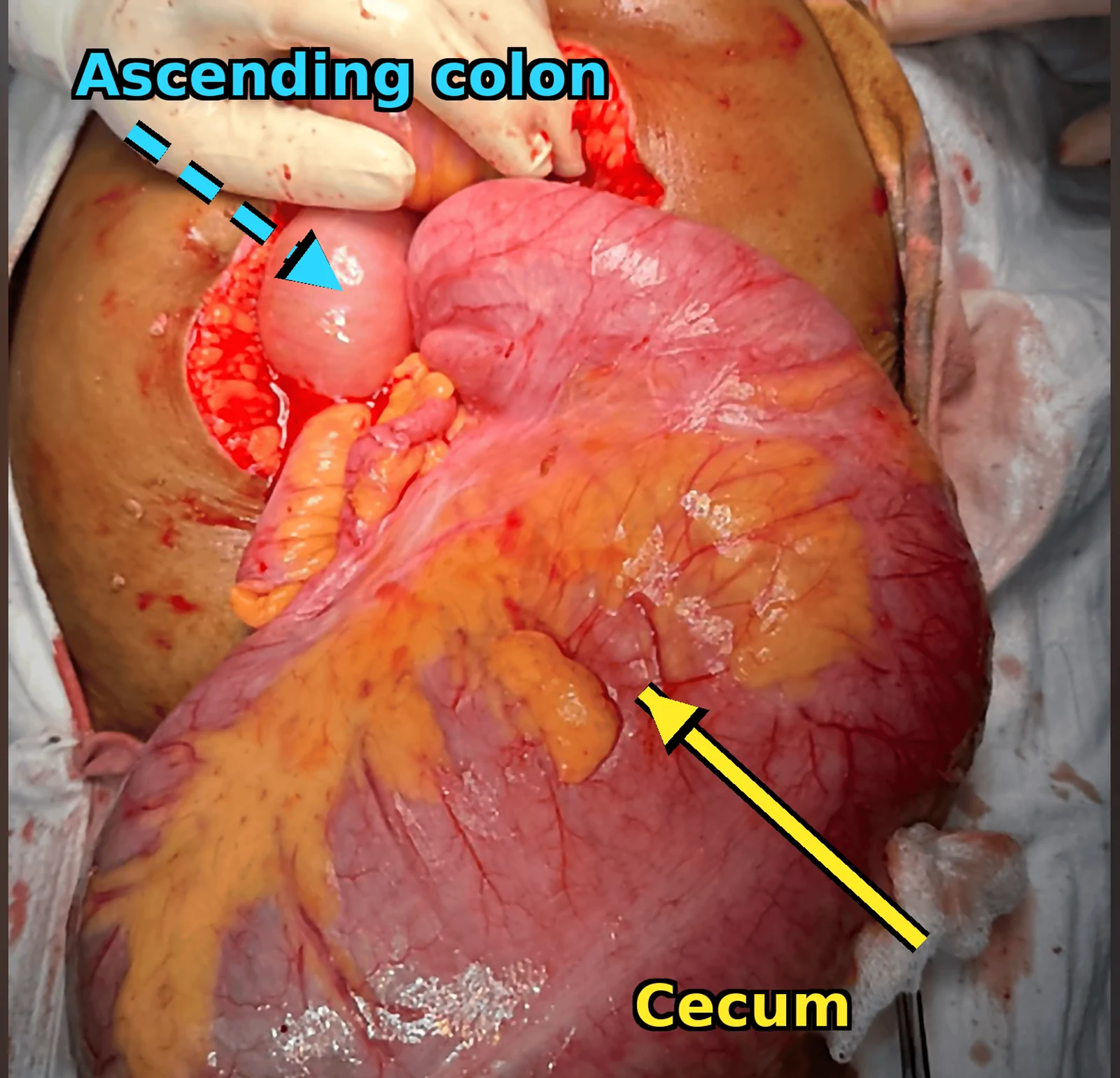
Intraoperative view showing marked distension of the caecum and ascending colon A massively distended cecum and ascending colon delivered through the incision; proximal small-bowel loops are visible. Solid arrow: distended cecum; dashed arrow: continuous ascending colon

**Figure 3:**
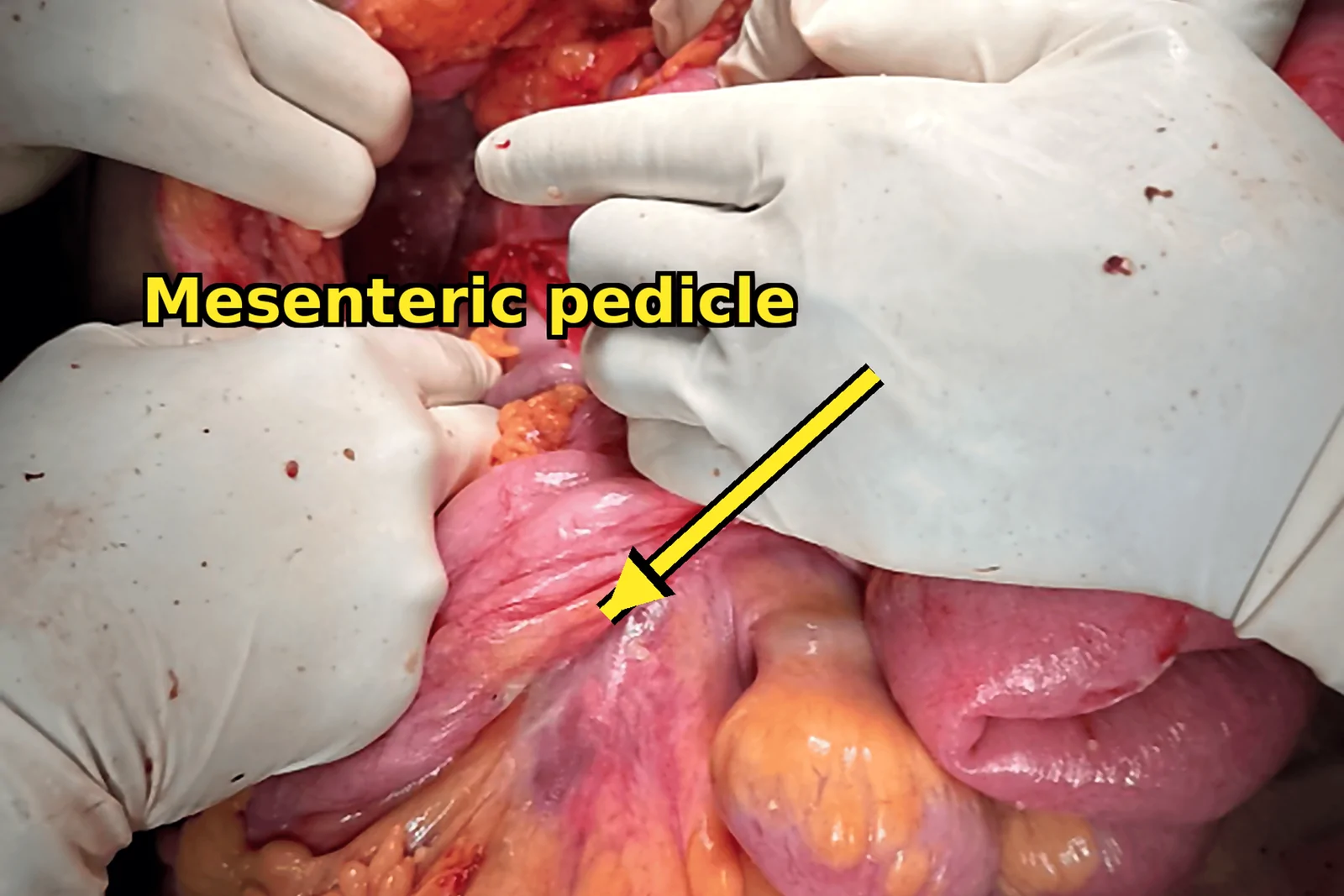
Intraoperative view showing the twisted mesenteric pedicle Distended, congested bowel loops with malrotation and a twisted mesentery; the surgical team is identifying anatomical structures and performing detorsion. Arrow: twisted mesenteric pedicle at the root of the volvulus

Following counterclockwise detorsion, viability was confirmed clinically by the return of pink color, visible peristalsis, and palpable mesenteric arterial pulsations; hand-held Doppler ultrasound was not required. Operative time was 115 minutes. The appearance of the cecum and ascending colon following detorsion is shown in Figure 4.

**Figure 4:**
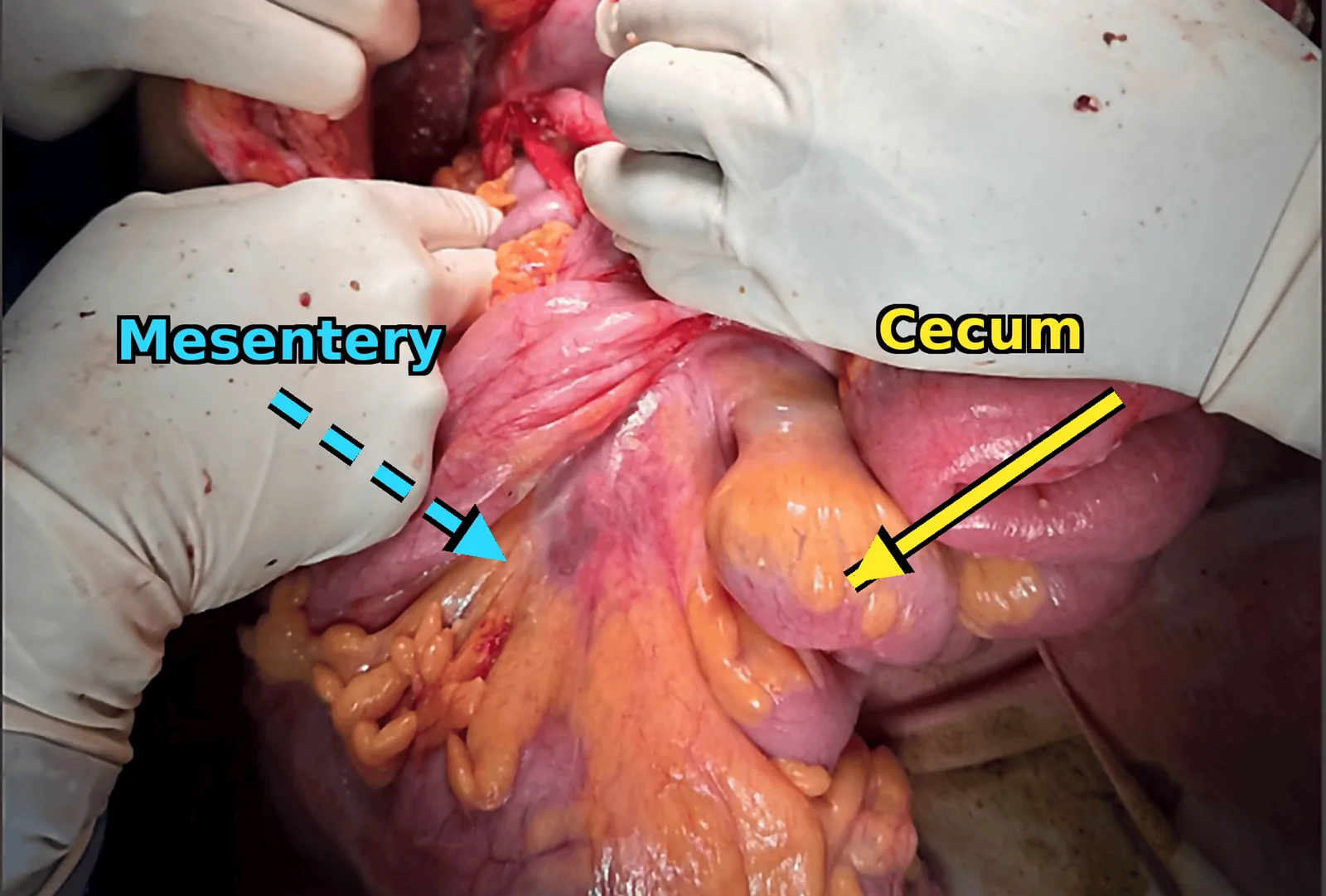
Intraoperative view showing the caecum and mesentery following detorsion Dilated cecum and ascending colon following detorsion. Solid arrow: detorsed cecum; dashed arrow: associated mesentery

Given that the bowel was viable and the perforation was single, small, and non-ischemic, a bowel-preserving strategy was chosen in preference to right hemicolectomy. The volvulus was detorsed, Ladd’s bands were divided to restore mesenteric mobility, and the colonic perforation was repaired primarily in two layers. Because of peritoneal contamination and to protect the suture line, a defunctioning loop ileostomy was fashioned proximal to the repair rather than performing a primary anastomosis in a contaminated field. Thorough peritoneal lavage was performed, and a pelvic drain was placed before closure. This approach avoided the loss of the ileocecal valve and ascending colon that hemicolectomy would entail, while diversion mitigated the risk of repair breakdown.

Postoperatively, the patient was managed with intravenous fluids, antibiotics, and graded diet advancement. The stoma began functioning on postoperative day three with appropriate output; oral intake resumed on day three and a soft diet was tolerated by day four; the drain was removed on day five, and she was discharged on day seven with a functioning ileostomy. The postoperative course was complicated only by a superficial surgical-site infection (Clavien-Dindo grade I), which resolved with local wound care. There was no clinical evidence of repair-site leak, dehiscence, recurrent perforation, or other repair-related complication. Histopathology showed acute inflammation with focal pressure/ischemic necrosis and no evidence of dysplasia or malignancy. The loop ileostomy was reversed at three months; at six months she was well, asymptomatic, and passing normal stool, with no clinical evidence of recurrence. The clinical timeline presented in Table 2 summarizes the patient's presentation, initial management, operative findings and treatment, postoperative recovery, discharge, ileostomy reversal, and six-month follow-up.

**Table 2:** Clinical timeline from presentation to six-month follow-up. ED, emergency department; IV, intravenous

Time point	Clinical course
Day 0 (presentation)	Emergency department: two-day history of abdominal pain, distension and absolute constipation; generalized peritonitis; resuscitation and antibiotics commenced.
Day 0	Erect radiograph demonstrated free gas beneath both hemidiaphragms. Emergency laparotomy was planned without cross-sectional imaging.
Day 0	Time from ED presentation to skin incision: 2.5 hours. Preoperative intravenous ceftriaxone 2 g and metronidazole 500 mg.
Day 0 (intraoperative)	Caecal volvulus with malrotation and Ladd’s bands; single anterior ascending-colon perforation. Detorsion, division of Ladd’s bands, primary two-layer repair, defunctioning loop ileostomy, peritoneal lavage, drain. Operative duration 115 minutes.
Days 1–5	Recovery on IV fluids and antibiotics; stoma functioning from day 3; oral intake resumed day 3, soft diet day 4; drain removed day 5.
Day 7	Discharged with a functioning ileostomy. Superficial surgical-site infection (Clavien–Dindo grade I) managed with local wound care. Histopathology: acute inflammation with focal pressure/ischemic necrosis; no dysplasia or malignancy.
3 months	Loop ileostomy reversed.
6 months	Well and asymptomatic with normal bowel habit; no clinical evidence of recurrence.

## Discussion

The literature cited in this case report was selected to provide relevant clinical and surgical context and was not derived from a systematic literature review. Intestinal malrotation is a rare congenital anomaly arising from incomplete rotation and fixation of the midgut. Although predominantly a pediatric diagnosis, it can present in adulthood with variable symptoms or as an acute abdominal emergency. Symptomatic adult malrotation is uncommon, and only a minority of affected adults present with volvulus or obstruction [1]. Rarer still is the coexistence of malrotation, cecal volvulus and colonic perforation, as seen here.

Our patient presented with absolute constipation, distension and radiographic pneumoperitoneum; intraoperative findings confirmed cecal volvulus with an ascending-colon perforation on a background of malrotation with Ladd’s bands. Unlike neonates, in whom malrotation typically manifests with bilious vomiting and failure to thrive, adults more often report chronic or intermittent pain, which contributes to diagnostic difficulty [2,3].

Mechanism and site of perforation

Although the title and discussion emphasize cecal volvulus, the perforation in this case was located in the ascending colon rather than the cecum. The most plausible mechanism is closed-loop obstruction from the volvulus, in which continued gas and fluid accumulation raises intraluminal pressure until wall tension exceeds perfusion pressure. By the law of Laplace, wall tension is greatest where the radius is largest; the resulting focal pressure necrosis and serosal tearing may occur at the point of maximal distension, which need not be the caecum itself. Localized venous congestion and microvascular compromise at this site - rather than global arterial ischemia - can therefore produce a single perforation while the remaining bowel stays macroscopically viable, as was observed. This reconciles the presence of a perforation with the preserved overall viability that permitted repair.

Several reports describe comparable presentations. Galaz et al. (2020) described a 25-year-old man with malrotation and midgut volvulus managed by emergency laparotomy and Ladd’s procedure [1]. Thomson et al. (2024) reported a 25-year-old woman with caecal volvulus and malrotation requiring right hemicolectomy, with incidental vascular anomalies [8]. Chaker et al. (2021) documented a 75-year-old man with cecal volvulus and malrotation treated by ileocecal resection and Ladd’s procedure [2], while Yahia et al. (2023) reported a 50-year-old man with an incomplete common mesentery and cecal volvulus with ischemia requiring resection and stoma formation [10]. Hashmi et al. (2020) described an 18-year-old with fecal peritonitis after cecal perforation managed by Ladd’s procedure and right hemicolectomy [9].

Comparison, differences, and lessons

A key difference between our case and several of the above is the intraoperative viability of the bowel. Where authors encountered ischemia, gangrene, or an unstable patient (e.g., Chaker, Yahia, and Hashmi), resection with or without stoma was appropriately performed. In our patient, the segment was congested but viable, and the perforation was single and small, which justified primary repair with proximal diversion rather than right hemicolectomy, preserving the ileocecal valve and ascending colon while protecting the repair. The use of a defunctioning ileostomy in the presence of contamination is consistent with the caution advocated by Yadav et al. (2017), who highlighted the role of fixation/diversion strategies in cecal volvulus with malrotation [7]. The principal lesson is that operative choice should be individualized to bowel viability and the degree of contamination rather than applied as a uniform resection.

In adults, the classic imaging features of malrotation include an abnormal duodenojejunal junction, right-sided small bowel, and inversion of the superior mesenteric artery-vein relationship, with a mesenteric whirl sign in volvulus [6]. Although CT is highly useful for establishing the diagnosis and defining the anatomical features of malrotation and volvulus in adults, its use should not delay definitive treatment in patients presenting with generalized peritonitis or pneumoperitoneum. In the present case, the radiographic evidence of perforation and clinical signs of generalized peritonitis prompted emergency laparotomy without CT, with malrotation and cecal volvulus confirmed intraoperatively.

Recurrence and long-term outcome

Long-term outcomes after treatment of intestinal malrotation in adults are generally favorable, although the available evidence is limited by the rarity of the condition and the predominance of retrospective series and case reports. In a series of 33 adolescent and adult patients treated with a Ladd’s procedure, postoperative complications were more frequent among patients requiring emergency surgery than among those undergoing elective repair (60% versus 22%), highlighting the greater morbidity associated with acute presentation [11]. Other small adult series have reported satisfactory symptom resolution after Ladd’s procedure, although postoperative complications, such as ileus or the need for reoperation, may occur [12]. Recurrent volvulus after Ladd’s procedure in adults has been reported but appears uncommon, and the true recurrence rate and optimal strategy for preventing recurrence remain uncertain because of the limited number of adult cases [13]. Bowel fixation procedures have therefore been considered in selected patients, particularly when a persistently narrow or mobile mesenteric base may predispose to recurrent volvulus [13]. In the present case, division of Ladd’s bands and restoration of mesenteric mobility were performed, and the patient remained asymptomatic without clinical evidence of recurrence at six months. Nevertheless, longer-term follow-up is warranted because the available evidence does not permit reliable prediction of recurrence or other late complications in adults.

Limitations

This is a single case, from which effectiveness cannot be inferred. Cross-sectional imaging was not obtained because urgent surgery was indicated, so radiological signs such as the whirl sign could not be documented; the available radiographs were photographed from a viewing monitor and, although de-identified and enhanced, are of limited resolution. Nonetheless, the completed histopathology and six-month clinical follow-up strengthen the report.

## Conclusions

Adult intestinal malrotation complicated by cecal volvulus and colonic perforation is rare and potentially life-threatening. Malrotation may remain silent until adulthood and present abruptly with obstruction or peritonitis, and the combination of Ladd’s bands, volvulus, and malposition poses both diagnostic and surgical challenges. In patients with generalized peritonitis and radiographic pneumoperitoneum, proceeding directly to laparotomy is appropriate, with the anatomical diagnosis confirmed intraoperatively. When the bowel is viable and the perforation is limited, primary repair with a defunctioning ileostomy is a reasonable, bowel-preserving alternative to resection. As a single case, this report cannot establish the superiority of any approach; rather, it illustrates that operative strategy should be tailored to intraoperative viability and contamination, and it emphasizes the value of documented follow-up, including ileostomy reversal.
